# A Cost-Effectiveness Analysis of the Swedish Universal Parenting Program All Children in Focus

**DOI:** 10.1371/journal.pone.0145201

**Published:** 2015-12-17

**Authors:** Malin Ulfsdotter, Lene Lindberg, Anna Månsdotter

**Affiliations:** 1 Department of Clinical Neuroscience, Karolinska Institutet, Stockholm, Sweden; 2 Department of Public Health Sciences, Karolinska Institutet, Stockholm, Sweden; London School of Hygiene and Tropical Medicine, UNITED KINGDOM

## Abstract

**Objective:**

There are few health economic evaluations of parenting programs with quality-adjusted life-years (QALYs) as the outcome measure. The objective of this study was, therefore, to conduct a cost-effectiveness analysis of the universal parenting program All Children in Focus (ABC). The goals were to estimate the costs of program implementation, investigate the health effects of the program, and examine its cost-effectiveness.

**Methods:**

A cost-effectiveness analysis was conducted. Costs included setup costs and operating costs. A parent proxy Visual Analog Scale was used to measure QALYs in children, whereas the General Health Questionnaire-12 was used for parents. A societal perspective was adopted, and the incremental cost-effectiveness ratio was calculated. To account for uncertainty in the estimate, the probability of cost-effectiveness was investigated, and sensitivity analyses were used to account for the uncertainty in cost data.

**Results:**

The cost was €326.3 per parent, of which €53.7 represented setup costs under the assumption that group leaders on average run 10 groups, and €272.6 was the operating costs. For health effects, the QALY gain was 0.0042 per child and 0.0027 per parent. These gains resulted in an incremental cost-effectiveness ratio for the base case of €47 290 per gained QALY. The sensitivity analyses resulted in ratios from €41 739 to €55 072. With the common Swedish threshold value of €55 000 per QALY, the probability of the ABC program being cost-effective was 50.8 percent.

**Conclusion:**

Our analysis of the ABC program demonstrates cost-effectiveness ratios below or just above the QALY threshold in Sweden. However, due to great uncertainty about the data, the health economic rationale for implementation should be further studied considering a longer time perspective, effects on siblings, and validated measuring techniques, before full scale implementation.

## Introduction

While knowledge regarding the effectiveness of parenting programs in terms of reduced child behavior problems and improved parenting has increased [1–4], knowledge of the economic value of these programs remains somewhat limited [5, 6]. In a review from 2014 [7], Stevens concludes that additional cost-effectiveness analyses of preventive family interventions are needed. Existing health economic analyses of parenting programs are mainly conducted on selective and indicated programs [8–12], where the aim is to reduce behavior problems in children. In studies by Edwards et al. [9] and O’Neill et al. [12], for example, cost-effectiveness analyses have been conducted to measure the cost per point reduction in the Eyberg Child Behavior Inventory (ECBI). O’Neill et al.[12] also include a cost-benefit analysis that includes future savings regarding education, crime, and unemployment. Furthermore, Sampaio et al. [13] use the ECBI to estimate cost-effectiveness for different parenting programs. The ECBI has also been used in modeling studies pertaining to reduction in clinical cases of conduct disorder [8, 10]. Another applied outcome in cost-effectiveness analysis is the Child Behavior Checklist (CBCL) [11], used to measure reduction in child behavior problems.

An issue with measures such as ECBI and CBCL is that problem behavior might not be the sole outcome that is important to study in parenting programs [14]. Another concern with using these outcome measures in cost-effectiveness analyses is that there is no established threshold value regarding society’s willingness-to-pay (WTP) for benefits on such scales. For example, the study by O’Neill et al. [12] resulted in an incremental cost-effectiveness ratio (ICER) of €87 per point reduction. This information may be used to guide resource allocation decisions between interventions with similar outcomes, but it does not allow comparison across interventions with other outcome measures [14]. For this reason, it is often suggested that a generic outcome measure is used, such as quality-adjusted life-years (QALYs). The use of QALYs is one of the key features of cost-utility analysis [14] and allows comparison between interventions that focus on any aspect of health. In addition, there is a generally accepted WTP for a unit of effect, i.e., for a gained QALY [15]. The QALY measure combines effects on health-related quality of life (HRQoL) on a scale from 0 to 1 with a period of time or with effects on premature mortality [16], e.g. the number of QALYs is 160 in a group of 20 people, with a HRQoL at 0.8, during 10 years (20 x 0.8 x 10). To our knowledge there is only one published study that has used QALYs to investigate the cost-effectiveness of a universal parenting program [17]. Simkiss et al. [17] used the SF-12 to derive QALYs gained among parents and concluded that the evaluated program was not cost-effective at the British WTP threshold value of £20 000–30 000 per gained QALY. Though, health economic evaluations of parenting programs, including the measurement of QALYs in children by parent proxies, are still lacking.

The universal parenting program “All Children in Focus” (the ABC program), is a universal parenting program offered to all parents with children aged 3–12 years [18]. The overall objective of the program is to promote parental competence and children’s positive development [18]. In the randomized controlled trial of the program, the main outcome measures were parental self-efficacy and children’s health and development, and the program was found to have a positive effect on both these outcomes [19]. Additionally, we intended to investigate if the program could be viewed as cost-effective and the overall objective of the present study is therefore to estimate the cost-effectiveness of the ABC program. We intend to estimate the costs associated with implementing the program, investigate the program’s health effects measured in QALYs in both children and parents, and, finally, to examine whether the program can be viewed as cost-effective.

## Methods

A cost-utility analysis (referred to as “cost-effectiveness analysis”) was performed from the societal perspective. The analysis considered the costs and effects of the intervention (the ABC program) compared to the control group (waiting list), and the incremental cost-effectiveness ratio (ICER) was calculated by the formula (C_1_-C_2_)/(E_1_-E_2_) [14]. Generally, the cost-effectiveness analysis was performed in order to answer if the question as to whether a future implementation of the program anywhere in Sweden would be a wise use of resources. This means that unit prices of the costs were collected alongside the trial, and then transformed to costs applicable for the national perspective. The WTP threshold for a QALY was set at 500 000 SEK (approximately €55 000) following a suggestion by the National Board of Health and Welfare [20].

### Study design

The ABC program was evaluated in a multicenter randomized controlled trial (Trial Registration Number: ISRCTN70202532). The trial and results on the primary outcome measures have been described elsewhere [18, 19]. Briefly, 621 parents in the County of Stockholm in Sweden agreed to participate in the trial and gave written informed consent. Parents were randomized to the intervention (n = 323), meaning that they received the program directly, or were assigned to a control group (n = 298). Control group parents were put on a waiting list and received the program after approximately six months. Parents in the intervention and control groups filled in questionnaires at baseline (before randomization), post-intervention (two weeks after the intervention ended for the intervention group), and follow-up (six months post baseline). The response rate at the post-intervention measurement was 92 percent, whereas 82 percent of the parents filled in the follow-up measurement. Alongside the trial, cost data were collected to enable the cost-effectiveness analysis. The study design was approved by the Regional Ethical Review Board in Stockholm (Dnr: 2012/93-31/5).

### Intervention

The ABC program is a universal program, which means that it is offered to the general population, including parents and/or their children with and without health problems [21]. One justification for universal public health interventions is the so-called prevention paradox, i.e., small effects at the individual level may have large effects at population level [22]. Universal interventions may also be more suitable for promoting positive aspects of health, and for decreasing the risk of stigmatizing vulnerable individuals in various aspects [18]. The ABC program consisted of four sessions and a booster session offered after 3–4 months. Each session lasted 2.5 hours, and the content of the sessions has been described elsewhere [18]. Parents received a binder which included materials for each session. Two trained group leaders ran the group, which contained at most ten parents. Within the trial, the average number of parents in a group was seven (Mean: 7.2, SD: 2.7). To date, the program has been found to promote parental self-efficacy and parents’ perceptions of their children’s health and development [19].

### Cost data

Several sources of data were used for the cost calculation, and costs were divided into setup and operating costs. Information about costs came from the ABC program developers, the literature, and group leaders, as described below. The costs were presented in the price level of 2014 using the Swedish consumer price index [23], and transformed from Swedish currency to Euro using the average exchange rate of 2014 (1 EURO = 9.1 SEK).

#### Setup costs

Setup costs included training in the program and time spent in training for group leaders. The cost for group leader training included fees for instructors (i.e., time and traveling), venues, material, refreshments during the days of training, and network meetings, as indicated by the price paid to be trained in the ABC program. Because group leaders run ABC groups within their ordinary occupation, i.e., they are not paid a fixed wage per group, it was deemed reasonable to estimate time costs for group leaders based on their ordinary hourly wage. A group leader’s time in training was therefore based on the spread of occupations among group leaders within the trial (including teachers, preschool teachers, parish assistants, psychologists, social workers, etc.), which was deemed representative of the national perspective. Information regarding their occupation was collected from a questionnaire to group leaders. Group leaders were initially informed orally and in writing about the trial and gave written informed consent concerning their participation (approved by the Regional Ethical Review Board in Stockholm, Dnr: 2012/93-31/5). The wage for each occupation was collected from national statistics (found for 66 group leader occupations of 68 in total) [24], and from there, an average hourly rate was estimated for group leaders including employee benefits of 31.42 percent [25]. The training had a total duration of 31 hours (in five sessions).

All setup costs were incurred during the first year of group leader training. However, in the analysis, these costs were assumed to be distributed over several groups. Because the ABC program is still fairly new, there are no available statistics on how many groups a group leader normally runs. At present, we know that few group leaders have run ten groups or more, but building on the assumption that current group leaders will be active for several more years, the average number was set at ten groups.

#### Operating costs

Operating costs were divided into municipal and societal costs. Municipal costs included group leader time spent on recruiting parents and holding the sessions, and the cost of venues, materials, and refreshments. The societal costs included parents’ time in sessions and travel costs.

Multiple channels, such as letters, web pages, phone calls, and school visits, were used to recruit parents. It was assumed that three hours on average was generally needed to fill one ABC group, approximately 25 minutes per parent. Group leaders were assumed to use four hours for preparing, running, and closing one session, i.e., 20 hours for one ABC group, which was supported by information from the program developers. The unit cost for venues was found in the literature [26], while the time needed per session was set at four hours (i.e., equal to the group leaders’ time for each session). The cost of materials for parents was given by the program developers. Furthermore, group leaders were according to the program manual assumed to provide refreshments for the parents during the sessions. The costs for refreshments at the sessions were based on the assumption that parents were offered coffee/tea and light refreshments.

Parents’ time at sessions was counted as their leisure time, which may be estimated differently. One approach has been to comprise this value in the numerator of the cost-effectiveness analysis, i.e., by using a percentage of the gross wage [27] or by asking individuals how much money they would pay for an additional hour of leisure time [28]. Another approach has been to incorporate leisure time in the denominator, i.e., by including it in the measurement of HRQoL [29]. Hagberg et al. [30] have found that people who were more experienced exercisers valued time spent on exercise as being equal to 7 percent of net wages, whereas less frequent exercisers valued the time to 26 percent of net wages. In an earlier study in the same field, Hatziandreu et al. [31] used 100 percent of the wage as the time cost for people who disliked exercise, 50 percent for those “neutral” (i.e., neither disliked, nor enjoyed) to exercise, and 0 percent for those who enjoyed exercise. It is possible to assume that parents who chose to participate, and also continued their participation (87 percent participated in two or more sessions), received some gratification so that the time cost would be rather low. Because there are no empirical studies on the valuation of parents’ time regarding participation in parenting programs, we choose to use a mean from the studies by Hagberg et al. [30] and Hatziandreu et al. [31]. Furthermore, we choose to double the lower values (0 and 7 percent) compared to the higher values (26, 50, and 100 percent), which resulted in the use of 27 percent of the average net wage in Sweden. The amount of time was based on parents’ attendance within the trial. A majority of the parents attended three or four of the sessions (i.e., 253 of 323); more specifically, 52.6 percent (n = 170) of the parents attended in all four sessions, 25.7 percent (n = 83) in three of the sessions, 8.7 percent (n = 28) in two of the sessions, 1.5 percent (n = 5) in one session, and 11.5 percent (n = 37) in none of the sessions [19]. At the booster session, 38.8 percent of the parents participated (based on 307 of the parents because attendance lists from the booster session were lacking for 10 parents).

During the trial of the program, most parents participated in an ABC group in their surrounding area. To represent a national perspective of travel costs, we assumed that parents needed one hour for traveling to and from each session. Furthermore, because there was no specified information concerning parents’ type of transportation to the sessions, we assumed that every other parent traveled by car (10 kilometer per session). The traveling time of the parents was valued as their leisure time, i.e., 27 percent of the national net wage according to estimation presented above.

### Effect data

#### QALYs in children by parent proxies

A parent proxy Visual Analog Scale (VAS) [14] was used within the trial to measure HRQoL in children, which was converted to indicate utility weights. The choice of using parents’ perceptions was based on the fact that the ages of the children ranged from 3 to 12 years [18], with a mean age at 6.09 years (SD 2.6) and with 71.5% being below 8 years of age. Eiser and Morse [32] have suggested that there may be no other option than to use proxy raters, which has been justified by the lack of cognitive skills in children that are too young [33]. Additionally, a piloting study among the ABC target population demonstrated difficulties as regards collecting questionnaires from children aged 3–12 years, where only 3 of 19 questionnaires were returned to us. In our trial, parents received a horizontal VAS with the following text: *Your child’s general health state today*. *The endpoints on the line represent the worst and the best possible health states that you can think of for your child*. *The worst health state is represented by 0 on the line*, *and the best health state is represented by 100*. *Estimate how good or bad your child’s general health state is between 0–100 in the box*. The scale was inspired by the EuroQol Group’s VAS (EQ-VAS) [34] for children [35, 36], but it was adjusted after a pilot study to fit within the trial of the ABC program. Our adjusted scale was a horizontal line instead of a vertical line, with no intervals marked out along the line. Further, the parents were asked to fill in the health state of their child in a box instead of marking it on the line. In health economic evaluations, it is valuable to have the health state values on the scale 0–1 where 1 equals to states of perfect health and 0 equals to states consistent with death [37]. With the formula, (raw rating of a health state − raw rating of death) / (raw rating of best health state − raw rating of death), health state valuations can be transformed and result in values on the 0–1 scale [37]. Since we had no ethical permission to ask parents to value perfect health or death on the VAS, we assumed 0 to represent death and 100 to represent perfect health. The parents’ raw ratings of their children’s health states were therefore divided by 100, i.e., to receive a value between 0–1 according to the above mentioned formula. The QALY change was derived by taking the mean of the two measurement periods (baseline mean + post-intervention mean / 2 vs. post-intervention mean + follow-up mean / 2) and multiplying it by 0.25 because each measurement period represented three months (i.e., 0.25 of a year) [15]. The calculation was based on the assumption of a linear growth trajectory, and to determine the incremental effect, the QALY change of the control group was subtracted from the QALY change of the intervention group. Concerning missing values, 5.4 percent of the VAS data was missing at baseline, 13.3 percent was missing at post-measurement, and 20.2 percent was missing at the follow-up measurement.

#### QALYs in parents

The General Health Questionnaire-12 (GHQ-12) [38] was used to assess mental health in parents, and was then further converted to indicate utility weights according to the proposal by Serrano-Aguilar et al. [39]. Our rationale to use the GHQ-12 was that it was the best measure available from the data collected in the trial that could be used for revealing QALY gains in parents. The parental questionnaire was tested in pilot studies and in order to optimize the response rate we had to restrict the number of questions. Hence, GHQ-12 was chosen as both a measure of mental health and the basis for utility weights. According to Goldberg and Williams [38], who developed the GHQ-12, it is a measure of mental health problems in community settings although not a diagnostic one, which was deemed suitable for the purpose and population in the ABC program. Concerning the contradiction in using both the concept of general health, i.e., the General Health Questionnaire, and assessing mental health, this emanates from the origin of the questionnaire [38]. GHQ-12 includes 12 items—six positively and six negatively phrased—answered on a four-category Likert scale [38]. To enable the conversion to utility weights, the scores were coded as 0 and 1. Category 1 (better/more so than usual, not at all) and category 2 (same as usual, no more than usual) were given the value 0, whereas category 3 (less than usual, rather more than usual) and category 4 (much less than usual, much more than usual) were given a value of 1. In all, the floor-maximum levels for the GHQ-12 were 0–12, whereas the end points for the utility weights were from worst to best imaginable health states, i.e. 0–1. The incremental QALY effect on parents was derived in the same way as the QALY calculation for children. Regarding missing values for the total GHQ-12 score, 2 percent were missing at baseline, 7.5 percent at post-measurement, and 17.3 at the follow-up measurement.

### Statistics

To examine differences between the intervention and control groups, and differences between baseline, post-intervention, and follow-up measurements within groups, independent and paired-samples *t*-tests were conducted in IBM SPSS Statistics for Windows, Version 22 (IBM Corp, Armonk, NY). The measurement of effects was based on intention-to-treat analysis, with the Last Observation Carried Forward (LOCF) method used to substitute for missing data regarding the VAS and GHQ-12 responses. Outliers represented by the converted values of 0.35 and below were removed regarding the VAS, which implied the lowest and highest individual utility weight values at 0.40 and 1.0 respectively. The outliers were also included in a calculation of QALYs in children by parent proxies to illustrate an ICER containing outliers. Regarding GHQ-12, the converted lowest and highest individual utility weight values ranged from 0.49 to 0.88 respectively.

### Probability- and sensitivity analyses

The probability of cost-effectiveness, which aimed at illustrating uncertainty due to sampling variation, was investigated in a cost-effectiveness acceptability curve (CEAC). The method of non-parametric bootstrapping was applied using EXCEL [40], where individual data were used to evaluate effects, whereas the mean was used for the costs. The result of the 5000 bootstrapped replicates was illustrated in the CEAC, in which the probability that the ABC program is cost-effective was mapped against different WTP levels. To retrieve the CEAC, the net monetary benefit method was applied, where the health effects were replaced with the WTP for a QALY [14].

The sensitivity analysis was univariate, i.e. based on varying essential variables one by one, and investigated the uncertainty in the following costs:

-*The distribution of the cost of group leader training*. In our base case, the setup cost was spread out over ten ABC groups. In the sensitivity analysis, the distribution was varied over five, fifteen, and twenty groups.
-*The cost of child care*. In our base case, no child care costs were included because only three of eleven municipalities/city districts offered child care to parents during the sessions within the trial. In the sensitivity analysis, we included the cost of a child sitter based on an hourly wage including employee benefits and the assumption of three hours per session (i.e., 15 hours at a time cost of €22.9 per hour).
-*The value of parents’ leisure time*. In our base case, the cost of parents’ leisure time was based on a weighted proportion of 27 percent of the net wage. In the sensitivity analysis, we employed 0 and 50 percent of the net wage to illustrate two cases: first, parents valued their participation in sessions markedly higher than the activity they sacrificed (represented by no cost), and secondly, parents valued their participation quite similar to the activity they sacrificed (represented by half the net wage).

## Results

### Costs

The setup cost to train a group leader was €1933. Based on the assumption that this cost was distributed over ten groups, the cost per ABC group was €386.6, i.e., total setup cost per group leader divided by ten groups and multiplied by two group leaders, or €53.7 per parent. See Table 1 for an overview of the setup costs.

**Table 1:** Setup costs for the ABC program.

Type of setup cost	Price per unit	Cost per group leader
Training fee	1 099 €/group leader	1099 €
Time (group leaders)	26.9 €/hour	834 €
**Total**		1933 €

The operating cost was €272.6 per parent or €1962.4 per ABC group, of which approximately 90 percent was paid by the municipality. See Table 2 for an overview of the operating costs.

**Table 2:** Operating costs for the ABC program.

Type of operating cost	Price per unit	Cost per ABC group	Cost per parent
**Municipal**			
Recruitment of parents	26.9 €/hour	80.7 €	11.2 €
Venue	11 €/hour	220 €	30.6 €
Group leader time	26.9 €/hour	1076 €	149.4 €
Material	7.8 €/binder	56.2 €	7.8 €
Refreshments	54.9 €/group	54.9 €	7.6 €
**Societal**			
Parents’ time	4.5 €/hour	275.9 €	38.3 €
Traveling (parents)			
Time	4.5 €/hour	162 €	22.5 €
Transportation	0.2 €/kilometer	36.7 €	5.1 €
**Total:**		1962.4 €	272.6 €

Hence, the total cost, including both setup and operating costs, was €326.3 per parent or €2349.4 for one ABC group.

### Effects

#### QALYs in children by parent proxies

There was no statistically significant difference in parents’ perceptions of children’s HRQoL by the VAS measure between the intervention and control groups at baseline, whereas there was a significant difference at the post-intervention measurement (*p* = 0.027) but not at the follow-up measurement (*p* = 0.479). Further, there was a significant increase between the baseline and post-intervention measurement for the intervention group (*t*[247] = -3.960, *p* < .001) and between the post-intervention and follow-up measurement for the control group (*t*[192] = -4.62, *p* < .001). See Table 3 for utility weights and statistical details.

**Table 3:** Utility weights in children by parent proxies (standard deviations) and *t*-statistics at baseline, post-intervention and follow-up, and QALY gains over the period from baseline to follow-up measurements (6 months).

	Means (SD)		*t*-statistics		
Measurement point	Intervention group*	Control group**	*t*	df	*p*-value
**Baseline**	0.8451 (.12)	0.8501 (.12)	-.455	472	0.656
**Post-measurement**	0.8709 (.11)	0.8479 (.12)	2.214	490	0.027
**Follow-up**	0.8702 (.11)	0.8770 (.10)	-.708	492	0.479
**QALY gains**	0.4323	0.4282	.900	472	0.369

*) Statistically significant within-group difference between baseline and post-intervention measurements (*p* < 0.001), but not between the post-intervention and follow-up measurements (*p* = .914).

**) Statistically non-significant within-group difference between baseline and post-intervention measurements (*p* = .945), but a statistically significant difference between the post-intervention and follow-up measurements (*p* < 0.001)

Children in the intervention group on average had a QALY change of 0.4321 ((((0.8451+0.8709)/2) + ((0.8709+0.8702)/2)) x 0.25) over the six month measurement period, while children in the control group on average had a QALY change of 0.4279 ((((0.8501+0.8479)/2) + ((0.8479+0.8770)/2)) x 0.25) over the same time period. This outcome resulted in a non-significant between-groups change of 0.0042 (0.4321–0.4279) QALYs gained per child (*t*[472] = .900, *p* = .369), in favor of the intervention group.

#### QALYs in parents

There was no statistically significant difference between the intervention and control group at baseline regarding parental utility weights. Nor were there any significant differences between the groups at the post-intervention measurement (*p* = 0.399) or follow-up measurement (*p* = 0.286). Regarding within-group differences, there was a significant increase from baseline to the post-intervention measurement for the intervention group (*t*[261] = -3.474, *p* < .01). See Table 4 for utility weights and statistical details.

**Table 4:** Utility weights in parents (standard deviations) and *t*-statistics at baseline, post-intervention and follow-up, and QALY gains over the period from baseline to follow-up measurements (6 months).

	Means (SD)		*t*-statistics		
Measurement point	Intervention group*	Control group**	*t*	df	*p*-value
**Baseline**	0.7761 (.13)	0.7839 (.13)	-.677	492	0.499
**Post-measurement**	0.8086 (.12)	0.7997 (.12)	.845	501	0.399
**Follow-up**	0.8099 (.12)	0.7985 (.12)	1.068	502	0.286
**QALY gains**	0.4003	0.3971	.766	492	0.444

*) Statistically significant within-group difference between baseline and post-intervention measurements (*p* = 0.001), but not between the post-intervention and follow-up measurements (*p* = .871).

**) Statistically non-significant within-group difference between baseline and post-intervention measurements (*p* = .131) or between post-intervention and follow-up measurements (*p* = .848).

Parents in the intervention group had an average QALY change of 0.4004 ((((0.7761+0.8086)/2) + ((0.8086+0.8099)/2)) x 0.25) over the measurement period whereas parents in the control group had an average QALY change of 0.3977 ((((0.7839+0.7997)/2) + ((0.7997+0.7985)/2)) x 0.25) over the same time period. This difference resulted in a non-significant between-groups change of 0.0027 (0.4004–0.3977) QALYs gained per parent (*t*[492] = .766, *p* = .444), in favor of the intervention group.

### Cost-effectiveness of the ABC program

**Fig 1:**
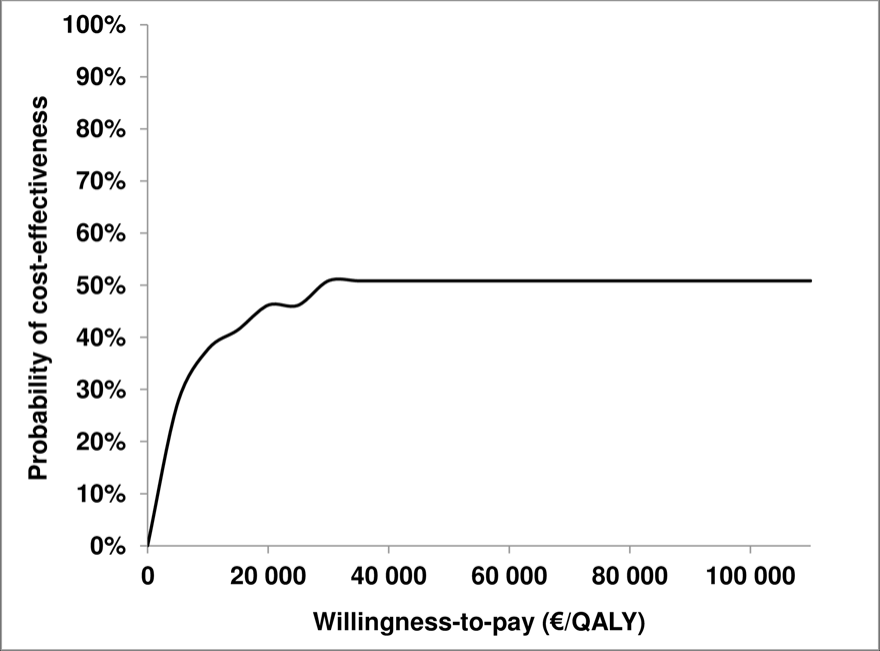
Cost-effectiveness acceptability curve (CEAC) showing probability for the program to be cost-effective at different WTP.

The total cost per parent was €326.3, and the total QALY gain for children as well as parents was 0.0069 (0.0042+0.0027). This outcome resulted in an ICER of €47 290 per QALY gained (326.3/0.0069). With outliers included in the calculation of QALYs in children by parent proxies, the ICER was €33 990 per QALY gained (326.3/(0.0069+0.0027)). The probability of the base case scenario being cost-effective at the threshold value €55 000 was 50.8 percent. See Fig 1 for the CEAC at different WTP thresholds.

Regarding the sensitivity analyses, variations of the costs resulted in ICERs below or just above the threshold value of €55 000, lying between €41 739 – €55 072. See Table 5 for results from the sensitivity analyses.

**Table 5:** ICERs from sensitivity analyses.

	Cost (€)/QALY
**Base case**	47 290
**Distribution of setup costs**	
**-5 groups**	55 072
**-15 groups**	44 696
**-20 groups**	43 391
**Childcare included**	54 203
**Parents’ leisure time**	
**-0%**	41 739
**-50%**	51 986

## Discussion

The cost-effectiveness analysis of the ABC program demonstrated an ICER of €47 290 per QALY gained. With the commonly applied Swedish WTP threshold value of €55 000 per QALY, the probability of the program being cost-effective was 50.8 percent. The univariate sensitivity analyses based on varying essential cost data showed that all ICERs (€41 739 – €55 072) were below or just above the QALY threshold. Because the probability of cost-effectiveness was around 50 percent from the WTP level at around €35 000, the ABC program (intervention) and the waiting list alternative (control) could be considered equally favorable for a decision-maker. The CEAC simply illustrates a probability of cost-effectiveness for different WTP thresholds [41], and the demonstrated point estimates from the base case and sensitivity analyses may therefore still be considered arguments for implementation. This position is strengthened from what is known as the payer perspective, rather than the applied societal perspective [14], since eliminating the costs for parents’ leisure time, for example, leads to an ICER below €42 000 per QALY. Nevertheless, this study is the first cost-effectiveness analysis of the ABC program, and further studies, particularly on long-term effects of the program, should be performed before recommendations are made for full scale implementation.

One rationale behind the present study is the lack of RCT-based effectiveness and cost-effectiveness analyses of universal parenting programs aimed at promoting health, rather than preventing disease. Generally, this means that the possibility to demonstrate large effects at the individual level is likely to be lower than for selected or indicated programs. However, since the decision-rule in cost-effectiveness analysis is health maximization considering limited resources, i.e. that interventions with small effects are desirable if the costs are small as well [14], universal programs may still have a health economic rationale. Our results could be compared with the cost-effectiveness analysis of the Family Links Nurturing Programme [17]. Although the conclusion was that this parenting program was not cost-effective for the British setting [42], the demonstrated ICER of £34 913 over 5 years (approximately €45 000) is similar to our ICER of €47 290 per QALY gained. However, it is important to consider in this comparison that Simkiss et al. [17] only calculated the QALY gains of parents and used the SF-12 measure, while we included the QALY gains of both children and parents and used other measures to retrieve QALYs. Furthermore, we applied a shorter time perspective of six months from baseline compared to nine months in the study by Simkiss and colleagues [17]. Even though effects of interventions generally decline over time, a short time perspective could mean that the positive effect of an intervention is not entirely measured, which could lead to a less favorable ICER. Since selected or indicated parenting programs often use outcomes linked to the reduction of behavior problems in children such as ECBI [8–10, 12] and CBCL [11], it is not possible to compare these studies with the present study. This leads us also to the issue of clinical relevance, which for health promotion tends to transform into salutogenic relevance. According to, for example [21], effect sizes for mental health promotion and prevention are usually small (Cohen’s d < 0.3) to moderate (Cohen’s d 0.3–0.7), while a clinically significant effect may correspond to a large effect (Cohen’s d > 0.8). A comprehensive understanding of clinical significance is further challenged in the present study by the fact that the VAS does not have a (well-established) clinical cut-off. Due to the general lack of normative data regarding the outcome measures used in the trial of the ABC program, it is also difficult to compare the study population to an average population. However, Burström et al. [36] have used EQ-VAS for children in 490 Swedish children (8 and 12 years old), where the mean VAS score was 89.1 compared with 84.8 in our analysis (mean of the intervention and control groups). Furthermore, according to the EQ-5D index in the Stockholm County population, the average utility weight among 34-44-year-olds was 0.85 compared with 0.78 in our analysis [43]. Parents recruited to the trial and their families thus seem less healthy compared to parents and children in general. However, because parents in the trial on average had higher education and income—attributes generally associated with good health—compared with the general population in the County of Stockholm, the differences are more likely to depend on the various measurement techniques.

Within this study, we were neither able to investigate how parents valued their time at the ABC sessions nor how they valued this time relative to the activity they sacrificed. Hence, the cost of parents’ time was derived from the time costs of exercise, another health-promoting activity [30, 31]. We know from the program developers that more than 95 percent of parents who participated in the ABC program would recommend it to other parents (based on a questionnaire given to 2777 parents with a response rate of 49 percent within the City of Stockholm). Furthermore, we know from an open-ended question given to parents within the trial that the majority of parents in the intervention group seemed to appreciate the program when expressing sentiments such as wanting all parents to be offered the program and being grateful for a rewarding program. In contrast, very few parents gave negative responses such as the sessions being too short or not divided appropriately for the age of the child. Similar results were found by Patterson et al. [44], where the majority of participating parents in the Webster-Stratton program had positive comments, whereas negative comments were few. The fact that most parents seemed satisfied with participating in parental programs led us to weight earlier findings on lower time costs more heavily than findings on higher time costs. Furthermore, even when the time cost of parents was set to 50 percent of the wage in the sensitivity analysis (compared to 27 percent in base case), the ICER was below the WTP threshold of €55 000 per QALY. Further studies are though needed to confirm our reasoning regarding the value of parents’ leisure time in parenting programs.

In the cost-effectiveness analysis, we also accepted non-significant mean differences of QALY gains, which may be questioned. However, according to Drummond et al. [14], it may not be appropriate to conclude that a non-significant difference is the same as a zero difference. As further explained by Glick et al. [15], there is a discussion within the economic community about sufficient evidence; more specifically, that the degree of confidence needed for health returns in economic evaluations could be lower than that of clinical outcomes in effectiveness studies. Claxton [45] has previously verified the importance of mean benefits, regardless of statistical significance. As proposed by Fenwick et al. [46], the uncertainty around point estimates may preferably be handled and illustrated by the CEAC. Based on the small effect found in the VAS outcome at the post-intervention measurement in this study, future power calculations regarding VAS for children should expect small effect sizes.

Regarding the mean parent proxy value of VAS, which was used to estimate QALYs in children, the control group had a higher mean (0.8770) compared to the intervention group (0.8702) at follow-up. A possible explanation is that for those parents on a waiting list for approximately 6 months, being aware that they would soon receive the intervention led them to make optimistically higher estimates regarding their own and their children’s health. This phenomenon of improvement in the control group, linked to beginning the ABC program, was also found in the case of the GHQ-12 measure, as well as observed for parental self-efficacy and children’s health and development, which have been studied previously [19]. However, these measures did not demonstrate that the control group had more beneficial estimates than the intervention group. The discriminative validity of EQ-VAS for children has been confirmed for children’s own reports in the age-groups eight and twelve years old [36]. That is, Burström and colleagues (2011) confirmed that groups of children, previously identified to differ in health status due to clinical and socio-economic characteristics, were distinguished also by scores of EQ-5D-Y, including the VAS [36]. Hence, generally, we suggest further investigation of the use of VAS as a measure for parents’ perceptions of HRQoL in children younger than eight years old and regarding other reliable measures of QALYs in children. This suggestion is confirmed by a recent systematic review by Thorrington and Eames [47], which concludes that measures of health utilities in children and adolescents are still being developed and validated, resulting in many studies using methods that have not been specifically designed for this age group, or segments of it.

One should also reflect upon the use of GHQ-12, via EQ-5D weights, into utility weights in parents by Serrano-Aguilar et al [39]. The most common instrument for estimating health gains in terms of QALYs is EQ-5D [48], which was considered in the present study. One reason for not applying this instrument was that the EQ-5D dimensions (mobility, self-care, usual activities, pain/discomfort, anxiety/depression) may fail to measure effects on the high levels of HRQoL in the target population of the ABC program. Additionally, as already mentioned, we wanted to restrict the amount of questions in the questionnaire, and therefore, the available method of converting GHQ-12 to utility weights [39] was deemed valid enough for the purpose of the present study.

As mentioned, the time perspective for the study was short, lasting only six months. Having a longer follow-up could have further benefited the cost-effectiveness of the ABC program. Such an extension could identify greater QALY gains from sustained HRQoL, as well as QALY gains and savings due to preventing problems from occurring, such as child behavior problems, school failure, etc. We thereby urge further cost-effectiveness analysis of the program, including potential consequences from a longer time perspective. Another issue to consider in future studies regards potential effects on siblings. In our trial, the QALYs gained were restricted to one child per parent, whereas the ABC program may have benefited other children in the family as well.

### Strengths and limitations

To date, there is a lack of health economic evaluations of universal parenting programs. In particular, studies including the measurement of QALYs are lacking, and to our knowledge, no study has included QALYs for both children and parents. Therefore, our study contributes to diminishing the knowledge gap regarding cost-effectiveness in this specific field. As a pioneering study, we also demonstrated the feasibility of reporting QALYs for both children and parents, despite earlier reported measurement challenges associated with QALYs in children [16]. Generally, our use of QALYs as an outcome measure enabled comparisons not only among parenting programs but also to other health interventions that are considered for implementation within the same budget.

However, the effect data on QALYs in children was based on parental proxies due to the young ages of a majority of the children that the ABC program targets (mean age 6 years), experiences from the piloting questionnaire, and the lack of well-established QALY measures for young children [16]. This may represent a concern in the present study. A review by Upton et al. [33] has explained that discrepancies between child and parent ratings occur, but also that parent proxies may be the only option when the children are unable to report their own values. Furthermore, they suggested that differences in the parents’ and children’s ratings are unlikely to be due to children’s ratings being more right and parents’ being more wrong but, rather that beliefs about HRQoL vary between individuals [33]. In a more recent review on the use of health utility measures among children and adolescents by Thorrington and Eames [47], the VAS measure and proxy perceptions were also evaluated. It was found that 22 direct or indirect methods were used a total of 137 times, of which VAS was used 14 times. Furthermore, 17 of the included 90 studies were exclusively based on parental proxies. Justification for the use of proxy respondents varied; several authors stated that proxy-reports may differ from self-reports, whilst others claimed that their use of proxies was in line with previous findings. In all, knowledge is still lacking regarding the validity of parents’ proxy assessments of utility in children [16]. In our case, however, the use of a parent proxy regarded exclusively children, and enabled us to include QALY gains of children, which seemed important for the health economic knowledge basis in the field of parenting programs.

Another concern regarding the measurement of QALYs in children by parent proxies is the VAS measure in itself, which has been questioned by some researchers [37, 49], whereas its potential has been highlighted by others [50]. The VAS is one of few direct measures of utility weights, but it can also be seen as second-best in comparison with the choice-based techniques of standard gamble (SG) and time trade-off (TTO) [37]. However, because we were in need of a simple and user-friendly measure for the parental questionnaire, the VAS was accepted as appropriate for our study. Regarding the VAS ratings, parents filled in a value between 0 and 100 in a box because the web-based questionnaire was not able to provide the possibility of making a cross or mark on the VAS. This limitation could have resulted in induced memory effects. However, it seems rather unlikely that parents would remember their ratings from three months before concerning a questionnaire of approximately 150 questions. Additionally, if the parents did remember, it would be unlikely to impact their rating. A final concern is that a full understanding is lacking about the fact that the control group had a higher mean compared to the intervention group at follow-up, i.e. when the former were about to begin the ABC program.

For parents, the GHQ-12 was used for conversion to utility weights, more specifically to EQ-5D weights. The relationship between the GHQ-12 and EQ-5D has been studied, and the model was found to show a high predictive capacity [39]. However, because there are several studies, and, hence, several data sets for both outcome measures, it seems reasonable to suggest further investigation of the validity of the relationship.

## Conclusions

Our analysis of the ABC program demonstrates cost-effectiveness ratios below or just above the Swedish WTP threshold of €55 000 per QALY gained. However, due to great uncertainty in the probability of cost-effectiveness, the health economic rationale for implementation is not yet convincing. Further analyses are needed to investigate the cost-effectiveness over a longer time perspective considering savings from prevention as well as health gains among siblings. We would also encourage further work regarding QALY measures in children for feasible health economic evaluations in the field of parenting programs.
